# Giant Cell Fibroma in a Two-Year-Old Child

**DOI:** 10.1155/2016/7058356

**Published:** 2016-10-16

**Authors:** Anna Carolina Volpi Mello-Moura, Ana Maria Antunes Santos, Gabriela Azevedo Vasconcelos Cunha Bonini, Cristina Giovannetti Del Conte Zardetto, Cacio Moura-Netto, Marcia Turolla Wanderley

**Affiliations:** ^1^Research and Clinical Center of Dental Trauma in Primary Teeth, Department of Orthodontics and Pediatric Dentistry, School of Dentistry, University of São Paulo-FOUSP, São Paulo, SP, Brazil; ^2^Ibirapuera University (UNIB) Master Program, São Paulo, SP, Brazil; ^3^Santa Cecília University (UNISANTA), Santos, SP, Brazil; ^4^Graduate Program in Dentistry, School of Dentistry, São Leopoldo Mandic, Campinas, SP, Brazil; ^5^Department of Orthodontics and Pediatric Dentistry, School of Dentistry, University of São Paulo-FOUSP, São Paulo, SP, Brazil; ^6^Clinical Oncology Service, Hospital Santa Catarina, São Paulo, SP, Brazil; ^7^Graduate Program in Dentistry, Cruzeiro do Sul University, São Paulo, SP, Brazil

## Abstract

The giant cell fibroma is a benign nonneoplastic fibrous tumor of the oral mucosa. It occurs in the first three decades of life in the mandibular gingiva, predominantly, showing predilection for females. This article reports a case of giant cell fibroma in a 2-year-old girl, which is an uncommon age for this lesion. The patient was brought for treatment at the Research and Clinical Center of Dental Trauma in Primary Teeth, where practice for the Discipline of Pediatric Dentistry (Faculty of Dentistry, University of São Paulo, Brazil) takes place. During clinical examination, a tissue growth was detected on the lingual gingival mucosa of the lower right primary incisors teeth. The lesion was excised under local anesthesia and submitted to histological examination at the Oral Pathology Department of the Faculty of Dentistry, University of São Paulo, which confirmed the diagnosis of giant cell fibroma. There was no recurrence after 20 months of monitoring. This instance reinforces the importance of oral care from the very first months of life in order to enable doctors to make precocious diagnosis and offer more appropriate treatments for oral diseases, as well as to promote more efficient oral health in the community.

## 1. Introduction

The giant cell fibroma is a nonneoplastic lesion with distinctive clinic-pathologic features [1]. The name “giant cell fibroma” has been assigned due to the presence of large stellate and multinucleated fibroblasts which are mainly in the lamina propria near the epithelium [1–4].

The giant cell fibroma usually occurs at young age, and it is more common in the second and third decades of life [5–7]. The prevalence is reported to be high in Caucasians with a slight female predilection [4, 8]. Lesions diagnosed in older people are likely to have already existed for many years [2]. Most cases predominantly occur on the mandibular gingiva [4, 8, 9]. However, the apex and lateral border of the tongue, buccal mucosa, palate, lip, and floor of the mouth are also common sites [2, 9, 10].

From a clinical perspective, the giant cell fibroma lesion appears as an asymptomatic pedunculated nodule with a papillary-like surface. The examined lesions were small, measuring less than 1 cm in diameter [1, 4, 11], frequently less than 0.5 cm [2–4, 10], which may cause them to be mistaken for a papilloma or gingival hyperplasia [11]. The consistency can vary from soft to firm [12]. It is a slow-growing lesion [10]. Histologically, the giant cell fibroma is an uncapsulated mass of loose fibrous connective tissue, noninflammatory, and covered with stratified squamous hyperplastic epithelium [1, 4, 11]. The conclusive diagnostic features of these lesions are the presence of large spindle-shaped, stellate-shaped, and mononuclear and multinucleated fibroblasts. The stellate cells showed large vesicular nuclei with prominent nucleoli. The cytoplasm of these cells was well demarcated and occasionally dendritic processes were observed. The cellular boundaries appeared to be separated from the surrounding collagen fibers in some areas and some of these cells contained melanin granules [1, 4, 11]. A prominent vascular element composed mainly of capillaries was also noticed [1, 4, 11]. Inflammatory processes rarely occur, unless the surface epithelium is ulcerated. When present, the inflammatory infiltrate is mono- and polimorfonuclear [1, 10, 11]. Giant cell fibroma could be diagnosed only on histopathological examination [5, 13].

The cause of giant cell fibroma is not well determined; however, some studies show that giant cell fibroma was considered as a response to trauma or recurrent chronic irritation and is characterized by functional changes in fibroblastic cells [14]. The treatment is surgical removal [4, 10, 12–15], and recurrence is rare [13].

This study reports a case of giant cell fibroma, located in the lingual gingival mucosa of the lower right primary incisors in a 2-year-old girl, and describes the main clinical and histologic findings, as well as the treatment.

## 2. Case Report

**Figure 1:**
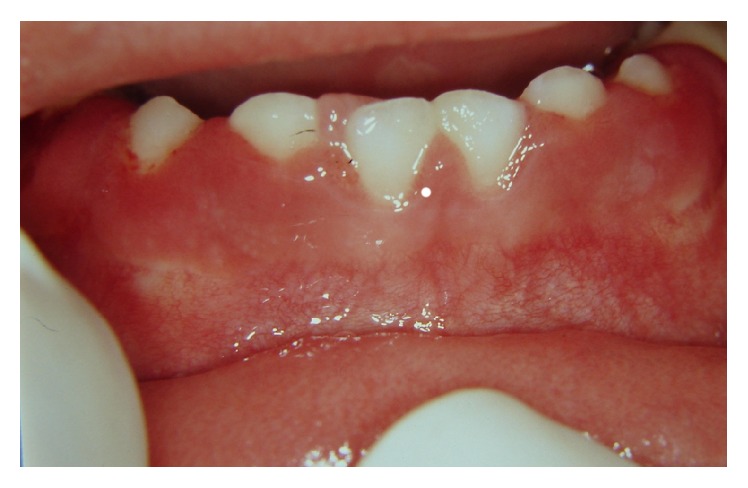
Frontal view of a tissue growth in the lingual gingiva next to the primary incisors in a 2-year-old girl. Note that gingivitis is present.

A 2-year-old Caucasian girl was brought for treatment at the Research and Clinical Center of Dental Trauma in Primary Teeth, where practice for the Discipline of Pediatric Dentistry (Faculty of Dentistry, University of São Paulo, Brazil) takes place. She presented total intrusion of the central lower right primary incisors. During clinical examination, tissue growth was detected on the lingual gingival mucosa of the lower right primary incisors teeth (Figure 1). According to the mother, the lesion was asymptomatic and she had not noticed it before. The child did not have any medical complications.

The patient was referred for treatment of traumatized primary tooth, as well oral preventive measures, since oral biofilm and gingivitis were present. The patient failed to attend the following session and returned only 6 months later.

**Figure 2:**
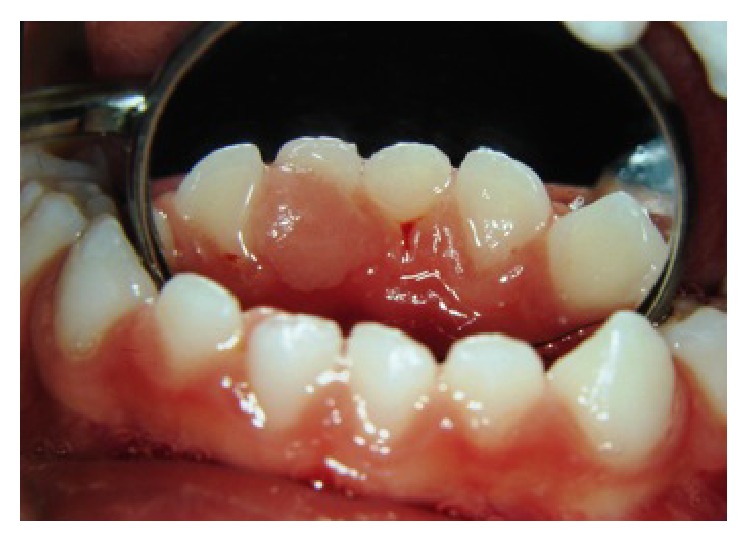
Lingual view of the gingival lesion. This image was shot six months after Figure 1.

**Figure 3:**
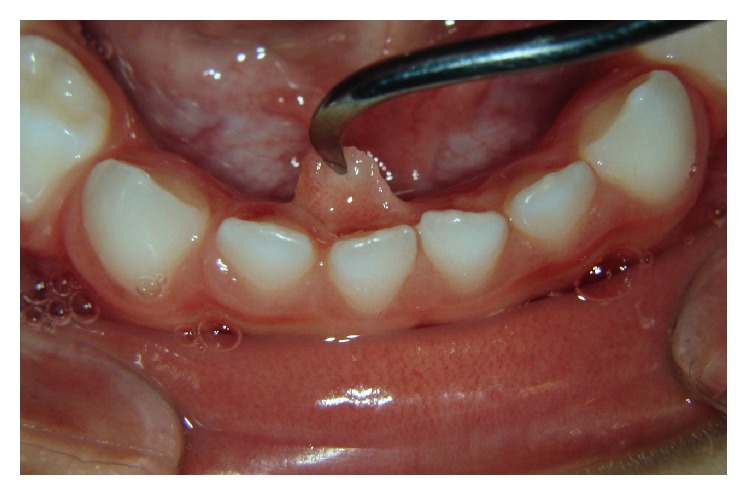
Pedunculated lesion in the lingual gingival mucosa, next to primary incisors.

On clinical examination, a pedunculated fibrous lesion was observed. This lesion was nonhemorrhagic of firm consistency and covered by intact white mucosa of approximately 5 mm × 5 mm × 3 mm in size. The primary teeth adjacent to the lesion maintained their original position. The lesion partially covered the lingual surface of the central and lateral lower right primary incisors. No other alteration was present in the oral cavity (Figures 2 and 3).

Based on the clinical appearance of the lesion, the differential diagnosis included primarily reactive and benign neoplastic lesions, such as fibroma, fibrous hyperplasia, peripheral ossifying fibroma, peripheral odontogenic fibroma, giant cell fibroma, and odontogenic hamartoma. As the procedure was simple, the lesion was excised under local anesthesia. The parents favoured less physical constraint; therefore, local anesthesia was used instead of general anesthesia. The young patient was sat on the dental chair. Her mother bent over her, holding her hands, whilst the assistant supported the child's head.

**Figure 4:**
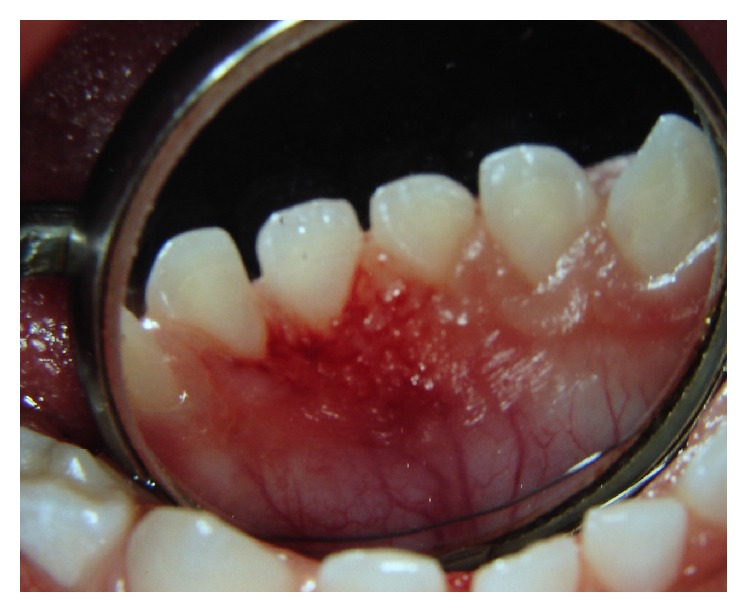
Appearance after surgical removal of the lesion.

A mouth prop was used to maintain adequate mouth opening. After topical anesthesia, local anesthesia was administered to the region of the incisors. Surgical removal was limited to the margins of the lesion in the gingival tissue. The excision was carried out using surgical scalpel blade number 15. Bleeding was very slight and was controlled using gaze compression. No suture was required (Figure 4).

**Figure 5:**
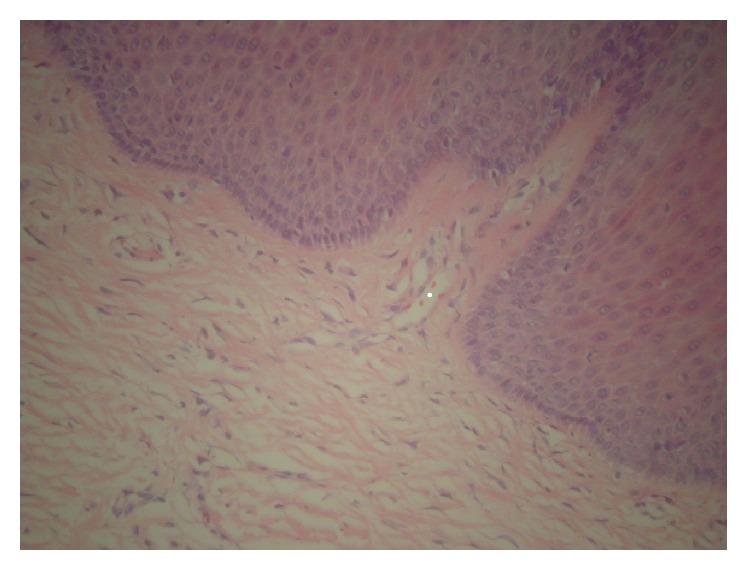
Scattered fibroblasts located just beneath the epithelium are enlarged and angular but are not hyperchromatic. Some cells have multiple nuclei. (H&E, original magnification 200x).

**Figure 6:**
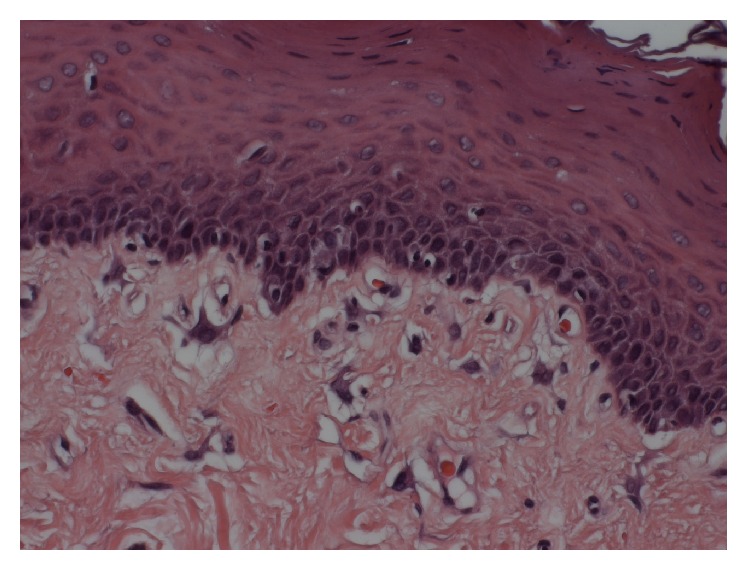
Slightly stellate multinucleated fibroblasts. (H&E, original magnification 400x).

Excised tissue was stored in 10% formaldehyde solution and submitted to the Oral Pathology Department of the Faculty of Dentistry, University of São Paulo, for histological analysis. The histological diagnosis revealed giant cell fibroma consisting of mucous tissue. This, in turn, was composed of parakeratinized stratified epithelium, which sent projections into the adjacent conjunctive tissue. Acanthosis, spongiosis, and exocytosis were also present. The lamina propria consisted of dense hyalinized connective tissue. Next to the juxta-epithelial hyalinization area, giant fibroblasts were found; they had two nuclei and stellate-form cells. In addition to the histological findings (Figures 5 and 6), extravasated red blood cells and often congested vascular spaces were found, which completed the findings.

**Figure 7:**
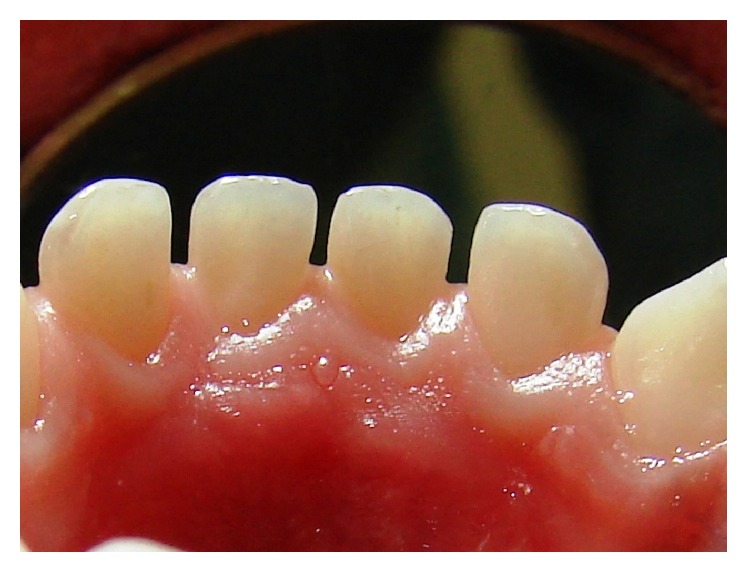
Clinical appearance after a 20-month follow-up.

No recurrence of the lesion was observed after a 20-month follow-up (Figure 7). The patient maintained good oral hygiene throughout this period.

## 3. Discussion

The clinical and histological features of the case we described were similar to the ones reported in the literature regarding giant cell fibroma. The giant cell fibroma is a benign fibrous tumor and it is most prevalent amongst the second and third decade of life [5–7], representing an average of 5% of all biopsied fibrous lesions [4, 9, 15] and around 1% of the total accessions in biopsy services [4, 9].

The differential diagnosis includes papilloma, fibroma, fibrous hyperplasia, and peripheral ossifying fibroma. The clinical aspects of these lesions are similar to those of the giant cell fibroma, such as the pedunculated nodule, the fibrous-looking papillary surface, and the ordinary colouration [1, 4, 11]. Despite this fact, the giant cell fibroma has its own particularities, that is, its own histological features and the prevalence of occurrence in certain (i) age groups, (ii) sex, and (iii) races, which makes it distinct from other lesions [1, 4, 5, 8, 11]. Last but not least, it should be noted that conducting a histopathological examination is essential to confirm the giant cell fibroma diagnosis [1, 5, 9, 11, 13].

Mono-, bi-, or multinucleated giant cells are not exclusive features of the giant cell fibroma, and they are detected in other lesions as well, such as ungual fibromas, acral angiofibroma, fibrous hyperplasia, and fibroma of the oral cavity [1, 7, 11].

The histopathological features found in this investigation are in agreement with the findings in the literature: benign fibroma showing mucous tissue, which are covered by parakeratinized stratified epithelium, projecting into a richly dense hyalinized connective tissue; such tissue is presenting many giant binucleated stellate-form fibroblasts [2–4, 10, 12, 16, 17].

The origin of the multinucleated and stellate cells in the giant cell fibroma is still unknown [18, 19]. It has been suggested that the mononuclear and multinucleated cells of giant cell fibroma may come from melanocytes or Langerhans cells [2, 4]. Histochemical studies have shown that these cells cannot be derived from the lineage of macrophages, monocytes, as they presented a negative reaction to CD68, LCA, and HLA-DR reagents [18, 19]. Other investigations have shown the fibroblastic origin of these cells. Campos and Gomez [18] suggested that the stellate and multinucleated cells of the giant cell fibroma are of fibroblastic origin and that there is certain difficulty in determining whether these cells undergo functional or degenerative changes [18].

Some authors reported that giant cell fibroma showed no predilection for gender [2–4], but other authors have indicated a preference for females [5, 6, 10, 12]. In this case report, the patient was a girl. Regarding race, literature has reported a marked predominance in Caucasians [2, 10], such as in this case report.

The giant cell fibroma usually occurs in young adults [10, 12, 14, 16, 20]. However, 5 to 15.5% of the evaluated giant cell fibroma was found in children from birth to 10 years of age [5, 8, 20]. Therefore, the case of a giant cell fibroma in a 2-year-old girl, as reported in this article, is a rare finding.

Regarding location, the lesion was found in the lingual gingival mucosa next to the lower right primary incisors, which confirms the information provided in the literature [2, 4, 8, 13, 20]. It is more commonly found in the mandible gingiva than in the maxilla (2 : 1) [2, 8, 13].

The clinical aspects of the lesion in this 2-year-old girl are similar to the ones reported by other authors [2, 4, 5, 10, 12, 13], as it presented a fibrous pedunculated asymptomatic characteristic; it was small, precisely less than 1 centimeter in size.

Recommendation was the complete surgical removal of the lesion, which is procedure that has commonly been suggested in other investigations [4, 5, 13, 15]. Total surgical removal was recommended in this case, as the lesion was very small. Furthermore, the lesion was detrimental to the child's oral hygiene. There was no recurrence of the lesion after a 20-month follow-up. Literature reports that recurrence is rare [4, 10, 12]. Houston only found 2 cases of recurrence in a total of 464 cases [4].

Children of very young age usually show resistance to dental treatment and, therefore, this is why diagnosis and treatment of oral lesions are not always made and offered at such an early age. In this case, the parents did not know their child had a lesion in her oral mucosa. This is probably due to the difficulty in examining baby's mouth and in identifying the exact time of eruption of the primary teeth, as well as others issues such as poor oral hygiene and presence of gingivitis. Oral and dental care should start at a very young age in order to enable dentists to provide effective guidelines for prevention and precocious diagnosis and enable them to offer adequate treatment of diseases of the oral cavity, thereby improving the chances of a correct prognosis and avoiding future problems.

## 4. Conclusion

It is very important that both oral and dental care begin at the very first months of life in order to promote oral health and enable doctors to make precocious diagnosis and offer more appropriate treatments for diseases of the oral cavity. In short, the clinical diagnosis of a giant cell fibroma in a 2-year-old girl was possible due to a thorough clinical examination. The lesion was asymptomatic and located in the lingual gingiva mucosa. The giant cell fibroma was successfully removed under local anesthesia. No recurrence was observed after a period of 20-months of monitoring.
